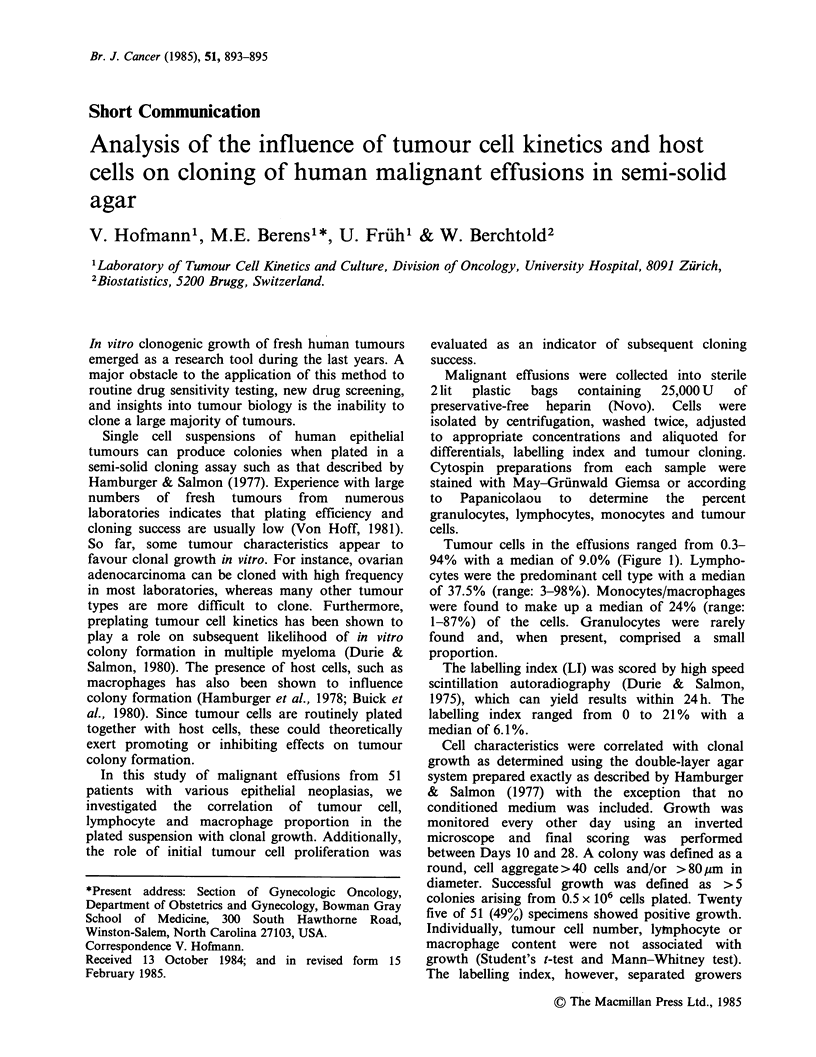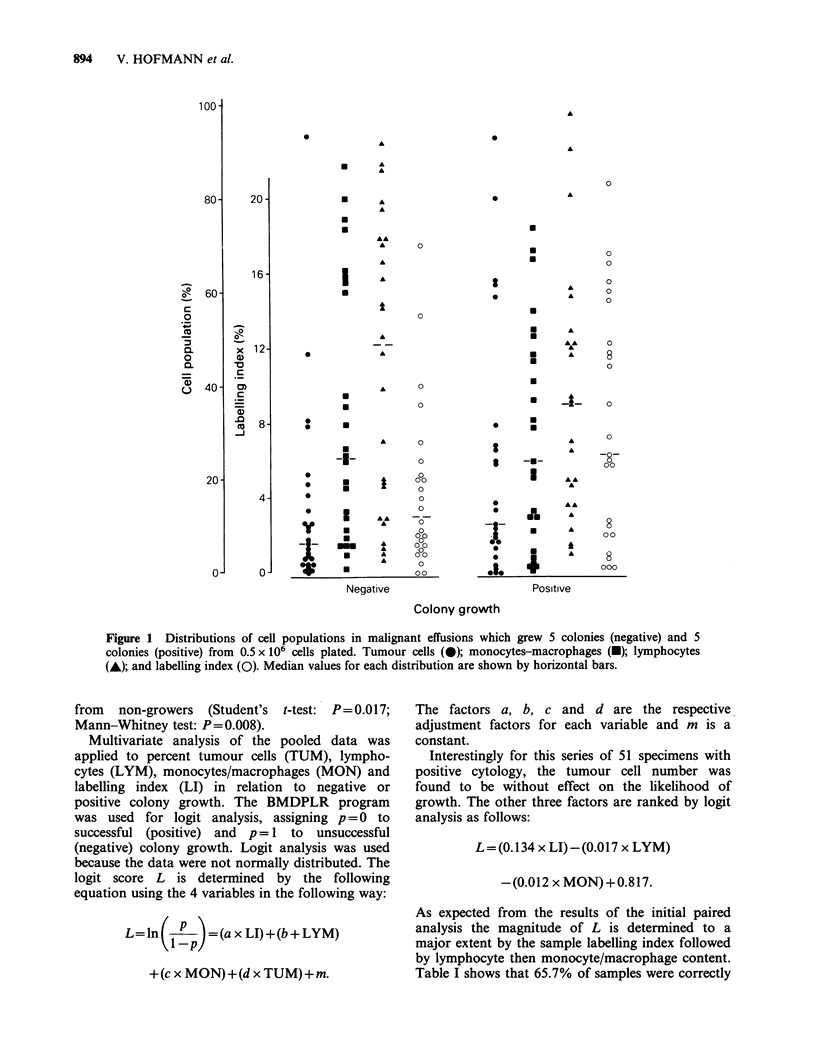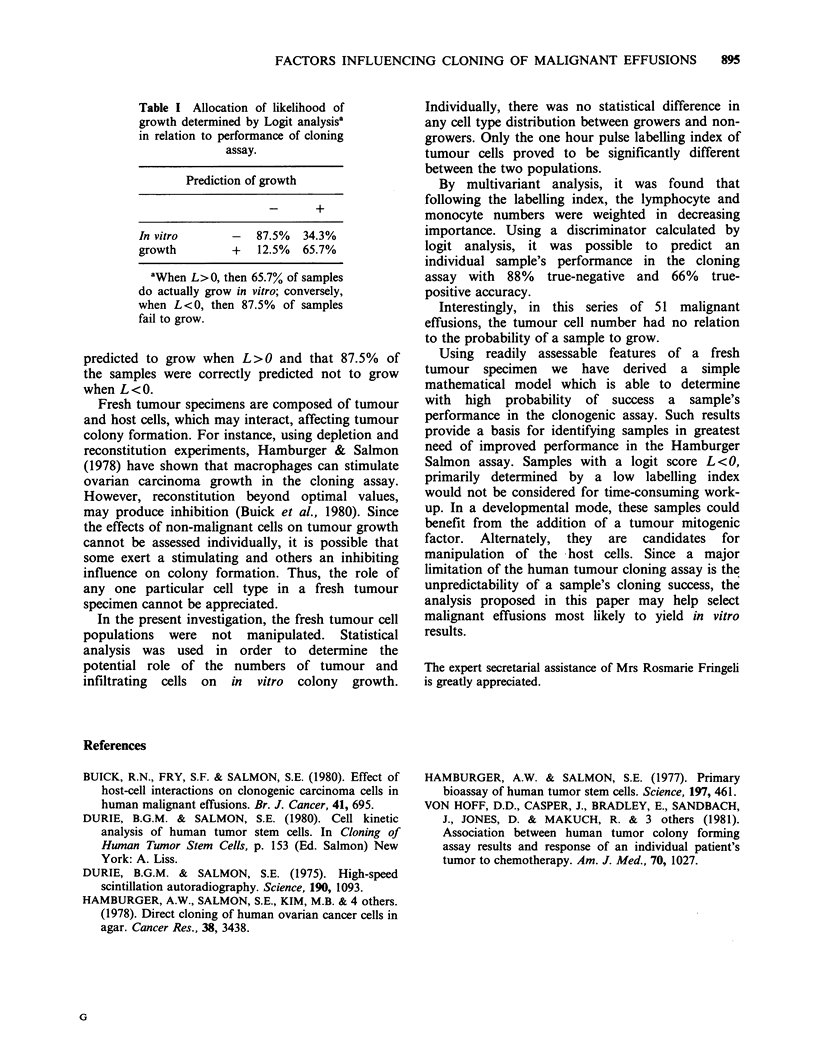# Analysis of the influence of tumour cell kinetics and host cells on cloning of human malignant effusions in semi-solid agar.

**DOI:** 10.1038/bjc.1985.135

**Published:** 1985-06

**Authors:** V. Hofmann, M. E. Berens, U. Früh, W. Berchtold


Br. J. Cancer (1985), 51, 893–895

### Short Communication

### Analysis of the influence of tumour cell kinetics and host cells on cloning of human malignant effusions in semi-solid agar

V. Hofmann[1], M.E. Berens[1]*, U. Früh[1] & W. Berchtold[2]

[1]*Laboratory of Tumour Cell Kinetics and Culture, Division of Oncology, University Hospital, 8091 Zürich,*
[2]*Biostatistics, 5200 Brugg, Switzerland.*

*In vitro* clonogenic growth of fresh human tumours emerged as a research tool during the last years. A major obstacle to the application of this method to routine drug sensitivity testing, new drug screening, and insights into tumour biology is the inability to clone a large majority of tumours.

Single cell suspensions of human epithelial tumours can produce colonies when plated in a semi-solid cloning assay such as that described by Hamburger & Salmon (1977). Experience with large numbers of fresh tumours from numerous laboratories indicates that plating efficiency and cloning success are usually low (Von Hoff, 1981). So far, some tumour characteristics appear to favour clonal growth *in vitro*. For instance, ovarian adenocarcinoma can be cloned with high frequency in most laboratories, whereas many other tumour types are more difficult to clone. Furthermore, preplating tumour cell kinetics has been shown to play a role on subsequent likelihood of *in vitro* colony formation in multiple myeloma (Durie & Salmon, 1980). The presence of host cells, such as macrophages has also been shown to influence colony formation (Hamburger *et al.*, 1978; Buick *et al.*, 1980). Since tumour cells are routinely plated together with host cells, these could theoretically exert promoting or inhibiting effects on tumour colony formation.

In this study of malignant effusions from 51 patients with various epithelial neoplasias, we investigated the correlation of tumour cell, lymphocyte and macrophage proportion in the plated suspension with clonal growth. Additionally, the role of initial tumour cell proliferation was evaluated as an indicator of subsequent cloning success.

Malignant effusions were collected into sterile 2 lit plastic bags containing 25,000 U of preservative-free heparin (Novo). Cells were isolated by centrifugation, washed twice, adjusted to appropriate concentrations and aliquoted for differentials, labelling index and tumour cloning. Cytospin preparations from each sample were stained with May–Grünwald Giemsa or according to Papanicolaou to determine the percent granulocytes, lymphocytes, monocytes and tumour cells.

Tumour cells in the effusions ranged from 0.3–94% with a median of 9.0% (Figure 1). Lymphocytes were the predominant cell type with a median of 37.5% (range: 3–98%). Monocytes/macrophages were found to make up a median of 24% (range: 1–87%) of the cells. Granulocytes were rarely found and, when present, comprised a small proportion.

The labelling index (LI) was scored by high speed scintillation autoradiography (Durie & Salmon, 1975), which can yield results within 24 h. The labelling index ranged from 0 to 21% with a median of 6.1%.

Cell characteristics were correlated with clonal growth as determined using the double-layer agar system prepared exactly as described by Hamburger & Salmon (1977) with the exception that no conditioned medium was included. Growth was monitored every other day using an inverted microscope and final scoring was performed between Days 10 and 28. A colony was defined as a round, cell aggregate > 40 cells and/or > 80 $\mu$m in diameter. Successful growth was defined as > 5 colonies arising from $0.5 \times 10^6$ cells plated. Twenty five of 51 (49%) specimens showed positive growth. Individually, tumour cell number, lymphocyte or macrophage content were not associated with growth (Student's *t*-test and Mann–Whitney test). The labelling index, however, separated growers

*Present address: Section of Gynecologic Oncology, Department of Obstetrics and Gynecology, Bowman Gray School of Medicine, 300 South Hawthorne Road, Winston-Salem, North Carolina 27103, USA.
Correspondence V. Hofmann.
Received 13 October 1984; and in revised form 15 February 1985.

© The Macmillan Press Ltd., 1985

**Figure 1** Distributions of cell populations in malignant effusions which grew 5 colonies (negative) and 5 colonies (positive) from $0.5 \times 10^6$ cells plated. Tumour cells (●); monocytes–macrophages (■); lymphocytes (▲); and labelling index (○). Median values for each distribution are shown by horizontal bars.

from non-growers (Student's *t*-test: $P=0.017$; Mann–Whitney test: $P=0.008$).

Multivariate analysis of the pooled data was applied to percent tumour cells (TUM), lymphocytes (LYM), monocytes/macrophages (MON) and labelling index (LI) in relation to negative or positive colony growth. The BMDPLR program was used for logit analysis, assigning $p=0$ to successful (positive) and $p=1$ to unsuccessful (negative) colony growth. Logit analysis was used because the data were not normally distributed. The logit score $L$ is determined by the following equation using the 4 variables in the following way:

$$L=\ln\left(\frac{p}{1-p}\right)=(a\times \mathrm{LI})+(b+\mathrm{LYM})$$
$$+(c\times \mathrm{MON})+(d\times \mathrm{TUM})+m.$$

The factors *a, b, c* and *d* are the respective adjustment factors for each variable and *m* is a constant.

Interestingly for this series of 51 specimens with positive cytology, the tumour cell number was found to be without effect on the likelihood of growth. The other three factors are ranked by logit analysis as follows:

$$L=(0.134\times \mathrm{LI})-(0.017\times \mathrm{LYM})$$
$$-(0.012\times \mathrm{MON})+0.817.$$

As expected from the results of the initial paired analysis the magnitude of $L$ is determined to a major extent by the sample labelling index followed by lymphocyte then monocyte/macrophage content. Table I shows that 65.7% of samples were correctly

**Table I** Allocation of likelihood of growth determined by Logit analysis[a] in relation to performance of cloning assay.

| | | Prediction of growth | |
|---|---|---|---|
| | | − | + |
| *In vitro* | − | 87.5% | 34.3% |
| growth | + | 12.5% | 65.7% |

[a]When $L>0$, then 65.7% of samples do actually grow *in vitro*; conversely, when $L<0$, then 87.5% of samples fail to grow.

predicted to grow when $L>0$ and that 87.5% of the samples were correctly predicted not to grow when $L<0$.

Fresh tumour specimens are composed of tumour and host cells, which may interact, affecting tumour colony formation. For instance, using depletion and reconstitution experiments, Hamburger & Salmon (1978) have shown that macrophages can stimulate ovarian carcinoma growth in the cloning assay. However, reconstitution beyond optimal values, may produce inhibition (Buick *et al.*, 1980). Since the effects of non-malignant cells on tumour growth cannot be assessed individually, it is possible that some exert a stimulating and others an inhibiting influence on colony formation. Thus, the role of any one particular cell type in a fresh tumour specimen cannot be appreciated.

In the present investigation, the fresh tumour cell populations were not manipulated. Statistical analysis was used in order to determine the potential role of the numbers of tumour and infiltrating cells on *in vitro* colony growth. Individually, there was no statistical difference in any cell type distribution between growers and non-growers. Only the one hour pulse labelling index of tumour cells proved to be significantly different between the two populations.

By multivariant analysis, it was found that following the labelling index, the lymphocyte and monocyte numbers were weighted in decreasing importance. Using a discriminator calculated by logit analysis, it was possible to predict an individual sample's performance in the cloning assay with 88% true-negative and 66% true-positive accuracy.

Interestingly, in this series of 51 malignant effusions, the tumour cell number had no relation to the probability of a sample to grow.

Using readily assessable features of a fresh tumour specimen we have derived a simple mathematical model which is able to determine with high probability of success a sample's performance in the clonogenic assay. Such results provide a basis for identifying samples in greatest need of improved performance in the Hamburger Salmon assay. Samples with a logit score $L<0$, primarily determined by a low labelling index would not be considered for time-consuming work-up. In a developmental mode, these samples could benefit from the addition of a tumour mitogenic factor. Alternately, they are candidates for manipulation of the host cells. Since a major limitation of the human tumour cloning assay is the unpredictability of a sample's cloning success, the analysis proposed in this paper may help select malignant effusions most likely to yield *in vitro* results.

The expert secretarial assistance of Mrs Rosmarie Fringeli is greatly appreciated.

### References

BUICK, R.N., FRY, S.F. & SALMON, S.E. (1980). Effect of host-cell interactions on clonogenic carcinoma cells in human malignant effusions. *Br. J. Cancer*, **41**, 695.

DURIE, B.G.M. & SALMON, S.E. (1980). Cell kinetic analysis of human tumor stem cells. In *Cloning of Human Tumor Stem Cells*, p. 153 (Ed. Salmon) New York: A. Liss.

DURIE, B.G.M. & SALMON, S.E. (1975). High-speed scintillation autoradiography. *Science*, **190**, 1093.

HAMBURGER, A.W., SALMON, S.E., KIM, M.B. & 4 others. (1978). Direct cloning of human ovarian cancer cells in agar. *Cancer Res.*, **38**, 3438.

HAMBURGER, A.W. & SALMON, S.E. (1977). Primary bioassay of human tumor stem cells. *Science*, **197**, 461.

VON HOFF, D.D., CASPER, J., BRADLEY, E., SANDBACH, J., JONES, D. & MAKUCH, R. & 3 others (1981). Association between human tumor colony forming assay results and response of an individual patient's tumor to chemotherapy. *Am. J. Med.*, **70**, 1027.